# Breathlessness and the body: Neuroimaging clues for the inferential leap

**DOI:** 10.1016/j.cortex.2017.07.019

**Published:** 2017-10

**Authors:** Olivia K. Faull, Anja Hayen, Kyle T.S. Pattinson

**Affiliations:** a FMRIB Centre, University of Oxford, Oxford, UK; b Nuffield Division of Anesthetics, Nuffield Department of Clinical Neurosciences, University of Oxford, Oxford, UK; c School of Psychology and Clinical Language Sciences, University of Reading, UK

**Keywords:** fMRI, Breathlessness, Symptoms, Anxiety sensitivity

## Abstract

Breathlessness debilitates millions of people with chronic illness. Mismatch between breathlessness severity and objective disease markers is common and poorly understood. Traditionally, sensory perception was conceptualised as a stimulus-response relationship, although this cannot explain how conditioned symptoms may occur in the absence of physiological signals from the lungs or airways. A Bayesian model is now proposed, in which the brain generates sensations based on expectations learnt from past experiences (priors), which are then checked against incoming afferent signals. In this model, psychological factors may act as moderators. They may alter priors, change the relative attention towards incoming sensory information, or alter comparisons between priors and sensations, leading to more variable interpretation of an equivalent afferent input.

In the present study we conducted a supplementary analysis of previously published data (Hayen et al., 2017). We hypothesised that individual differences in psychological traits (anxiety, depression, anxiety sensitivity) would correlate with the variability of subjective perceptions of equivalent breathlessness challenges. To better understand the resulting inferential leap in the brain, we explored where these behavioural measures correlated with functional brain activity across subjects.

Behaviourally, anxiety sensitivity was found to positively correlate with each subject's variability of intensity and unpleasantness during mild breathlessness, and with variability of unpleasantness during strong breathlessness. In the brain, anxiety sensitivity was found to negatively correlate with precuneus activity during anticipation, positively correlate with anterior insula activity during mild breathlessness, and negatively correlate with parietal sensorimotor areas during strong breathlessness.

Our findings suggest that anxiety sensitivity may reduce the robustness of this Bayesian sensory perception system, increasing the variability of breathlessness perception and possibly susceptibility to symptom misinterpretation. These preliminary findings in healthy individuals demonstrate how differences in psychological function influence the way we experience bodily sensations, which might direct us towards better understanding of symptom mismatch in clinical populations.

## 1 Introduction

*“If the doors of perception were cleansed everything would appear to man as it is, infinite.*

*For man has closed himself up till he sees all things thro’* *narrow chinks of his cavern.”*

WILLIAM BLAKE, *The Marriage of Heaven and Hell*

The perception of bodily sensation is integral to the management of self within the environment. One frightening and debilitating perception is that of breathlessness, when breathing is perceived as inadequate and a threat to life. Breathlessness is experienced across a range of illnesses (Smoller et al., 1996, Solano et al., 2006), including lung disease, heart disease and cancer. Breathlessness is notorious as a symptom that is often out of proportion to objective markers of disease (Hayen et al., 2013, Herigstad et al., 2011, Jones, 2001, Lansing et al., 2009, Mahler et al., 1996). While perceptual systems have traditionally been considered to encompass a stimulus followed by the brain's response, this relationship cannot explain the often-observed dissociation between perception and symptom extent, with extreme cases manifesting as medically unexplained symptoms (Isaac and Paauw, 2014, Nimnuan et al., 2001). As it is the perception of symptoms that leads to their debilitating consequences, an overhaul is required in the way we consider the brain's interaction with incoming sensory information. This would lead to better ways to understand and then treat unpleasant perceptions such as breathlessness.

With a launch into the Bayesian tidal wave of modern neuroscience (Colombo and Seriès, 2012, Friston et al., 2002, Geisler and Albrecht, 1995, Ma et al., 2006, Miyazaki et al., 2006), recent theories have proposed a comprehensive model of symptom perception (Barrett and Simmons, 2015, Van den Bergh et al., 2017). An important development of this model is the inclusion of a set of perceptual expectations, or ‘doors of perception’ in the words of William Blake. These perceptual ‘priors’ are neural representations of a distribution of expected values, which may be separated from the afferent neural inputs. Both priors and afferent sensory information can influence perception, which encompasses a range of probable perceptions (posterior distribution). Enhanced confidence in expectations (narrow, sharp priors) can increase their weight in the model, pulling the resulting perception away from the physiology and towards the prior. Furthermore, perceptual moderators exist within this system, such as anxiety (Bogaerts et al., 2005, Spinhoven et al., 1997, Tang and Gibson, 2005), attention (Ling and Carrasco, 2006, Merikle and Joordens, 1997, Phelps et al., 2006), arousal (Allen, Frank, Schwarzkopf, & Fardo, 2016) or interoceptive ability (Critchley et al., 2013, Garfinkel, Tiley et al., 2016, Gray et al., 2007, Mallorqui-Bague et al., 2016), which may adjust either the prior expectations or incoming sensory information to influence perception. Beyond symptom perception, these moderators may even directly influence sensory information, as previous research has linked anxiety and worry with greater variability in ventilatory patterns (Rainville et al., 2006, Vlemincx et al., 2010, Vlemincx et al., 2013). Lastly, individuals who more frequently report symptoms have been shown to be less accurate towards their ventilatory interoceptive perceptions (Bogaerts et al., 2008), suggesting a complex relationship between symptom awareness and accuracy of bodily sensations. For instance, perception may be shifted to be higher or lower than the sensation, or there may be a greater range of possible perception values (widened distribution), which increases their ambiguity and susceptibility to misinterpretation and misclassification as a potential threat.

The ‘inferential leap’ to reconcile expectation and neural sensory information and form conscious perception occurs in the brain (Allen and Friston, 2016, Van den Bergh et al., 2017). One seductive theory consists of a division between agranular cortices (such as the anterior cingulate cortex and anterior insula) that generate prediction signals, and granular cortices (such as the primary sensory cortex and posterior insula), which compare afferent signals with predictions to generate prediction errors (Barrett and Simmons, 2015, Friston, 2005, Geuter et al., 2017, Shipp et al., 2013). It is hypothesized that behavioural factors such as decreased or redirected attention could also reduce the gain of sensory information within granular cortices (Feldman & Friston, 2010), thereby diminishing the prediction error by increasing the relative weight of the priors in the model (Barrett and Simmons, 2015, Feldman and Friston, 2010). Alternatively, behavioural influences may reduce the gain of the prior within agranular cortices (Barrett & Simmons, 2015) to reduce prediction errors and influence perception.

In this report we have firstly investigated whether behavioural scores of anxiety, depression and anxiety sensitivity relate to the distribution of subjective scores (posterior perceptual distribution) of experimentally induced breathlessness. Mild and strong breathlessness were indicated by a conditioned stimulus (a shape presented on a screen), and implemented after a short anticipation period. Both levels of breathlessness were considered, as sensory afferents may be more vague or indefinite during mild breathlessness stimuli and might thus rely more heavily on priors. Here, we have undertaken a supplementary analysis on previously unreported aspects of a recently published study of Hayen el al. (2017) to explore where in the brain these perceptual moderators act to alter perception.

## 2 Materials and methods

This study originally aimed to characterise functional brain activity during perception of conditioned mild and strong breathlessness stimuli in 19 healthy participants (10 females, mean age ± SD, 24 ± 7 years). An account of conditioned responses to strong breathlessness has been published previously (Hayen et al., 2017), where the mild breathlessness stimulus was not considered due to its large between-subject variability. In the current report we have undertaken a more detailed, exploratory, post-hoc evaluation of how behavioural measures relate to subjective stimulus perceptions in both the mild and strong conditions, and where in the brain these perceptions may be modulated. Please see Hayen et al. (2017) for a complete description of data acquisition and the lower level functional magnetic resonance imaging (fMRI) analysis. The study of Hayen et al. was a blinded placebo-controlled study of the effect of an opioid (remifentanil) on breathlessness, but in the present paper we only consider the placebo condition (infusion of .9% saline).

### 2.1 Participants

Written informed consent was obtained in 29 participants, in accordance with the Oxfordshire Research Ethics Committee. Data from 19 healthy participants (10 females, age 24 (±7 SD) years) was analysed, with 10 excluded for the following reasons: 2 participants exhibited vasovagal syncope during cannulation; 1 participant did not comply with study instructions; 4 participants did not learn the association between visual cues and respiratory stimuli; 3 participants were excluded because of technical difficulties with the MRI equipment. We only recruited female participants if they were taking the combined oral contraceptive pill to minimise any potential effects of hormonal cycle fluctuations (Rosendaal, Helmerhorst, & Vandenbroucke, 2001) upon study findings via altered ventilation (Das, 1998), cerebrovascular reactivity (Krejza, Rudzinski, Arkuszewski, Onuoha, & Melhem, 2013) and opioid efficacy (Terner, Lomas, & Picker, 2005). Participants were right-handed non-smokers that were generally healthy, not receiving any medication and had no history of neurological (including painful conditions), pulmonary or cardiovascular disease. They were free from clinical depression and anxiety disorders, and there were no reports of any previous depressive or anxious episodes of any kind in all participants.

Before the training session, participants were instructed to breathe normally, pay attention to the screen in front of them and rate their breathing intensity and unpleasantness when instructed. After the session, participants completed detailed feedback on their breathing experiences for each of the three conditions. Hence, participants were encouraged to think about their breathing in three distinct categories relating to the abstract shapes, which allows them to form priors to predict the next experience. Participants and researchers were fully blinded to the order of administration of saline and remifentanil.

### 2.2 Behavioural questionnaires

Depression was measured using the Center for Epidemiologic Studies Depression Scale (revised) (CESD-R (Radloff, 1977)). The trait scale of the Spielberger State-Trait Anxiety Inventory (STAI (Spielberger, 2010)) was used to characterize general participant anxiety. The Anxiety Sensitivity Index (ASI (Reiss, Peterson, Gursky, & McNally, 1986)) was used to differentiate sensitivity to symptoms of anxiety in the form of bodily perceptions.

### 2.3 Conditioned breathlessness and functional brain scanning

Scanning was conducted using a 3 T Siemens Trio scanner, with physiological monitoring and control of end-tidal gases (see Hayen et al., 2017). Briefly, an aversive delay-conditioning session was performed outside of the scanner, followed by two fMRI sessions on consecutive days (remifentanil or saline placebo, counterbalanced across participants). Participants learnt associations between three visual cues and three respiratory sensations during the conditioning session, which were mild breathlessness (mean ± SD: 4.0 ± .8 cmH_2_O), strong breathlessness (12.5 ± 4.1 cmH_2_O) or no breathlessness (unloaded breathing: 2.7 ± .7 cmH_2_O). The breathlessness stimulus used in this study was intermittent resistive inspiratory loading for 30–60 sec, administered via an MRI compatible breathing system (Hayen et al., 2017). Expiration was unrestricted via a one-way valve (Hans Rudolph, Shawnee, Kansas, USA). The stimuli were each presented four times during the scanning session in a semi-randomised, counterbalanced order, with a preceding anticipation period of 8 sec followed by a resistive loading stimulus (where appropriate). Immediately following each stimulus, participants were asked to rate both the intensity and unpleasantness of the preceding load on a visual analogue scale (VAS: 0–100%).

### 2.4 Behavioural and fMRI analysis

In this short report we only consider the fMRI session with the saline infusion. Full details on analysis procedures have been previously reported (Hayen et al., 2017), and involved robust physiological noise correction of fMRI images. Briefly, this included using independent component analysis (ICA (Griffanti et al., 2014, Griffanti et al., 2017, Salimi-Khorshidi et al., 2014)) to decompose and remove noise components from the data, followed by regression of the harmonics from respiratory and heart rate recording traces acquired during scanning (using FEAT's Physiological Noise Modeling tool, PNM (Brooks et al., 2013, Harvey et al., 2008)). Complete heart rate traces were only available in 15 subjects, however ICA noise correction (which removes much of the cardiac noise without needing a physiological trace) and PNM using only the respiratory trace were still performed on the remaining 4 subjects. Whilst former analyses examined mean brain responses to anticipation and breathlessness (and the changes induced by remifentanil), the focus of this analysis was to explore how behavioural measures relate to the mean and variability of breathlessness perceptions in each subject, and to any corresponding changes in brain activity.

Mean and variability (SD) of mouth pressure, subjective intensity and unpleasantness during scanning for both mild and strong loading were calculated for each subject (Table 1). A full exploratory correlation matrix was then created on all behavioural and physiological variables, including questionnaires, mouth pressure and subjective breathlessness scores for each level of loading. As the behavioural variable ASI score was shown to significantly correlate with trial-by-trial variation (SD) of subjective scores, the group fMRI analysis previously reported (Hayen et al., 2017) was adjusted to include a group mean and ASI score regressor, as well as covariates to exclude the effects of age, gender and the order of saline and remifentanil sessions for each subject. This analysis aimed to identify where functional brain activity correlates with differences in ASI score and thus extent of perceptual variability across subjects during saline administration, using the standard (arbitrary) cluster-forming Z threshold of 2.3, followed by whole-brain correction of these clusters for multiple comparisons (*p* = .05) using Gaussian Random Field theory in FSL (FMRIB's Software Library, www.fmrib.ox.ac.uk/fsl).

**Table 1:** Effects of loading on respiratory parameters. P_ET_CO_2_ = partial pressure of end-tidal carbon dioxide. P_ET_O_2_ = partial pressure of end-tidal oxygen. Values are presented as mean (SD). *N* = 19. Complete heart rate data in each epoch only available for 15 subjects.

Variable	Anticipation unloaded	Unloaded breathing	Anticipation mild	Mild loading	Anticipation strong	Strong loading
Mouth pressure amplitude [cmH_2_O]	2.7 (.7)	2.4 (.5)	2.6 (.7)	4.0 (.8)	3.5 (1.7)^¶^	12.7 (4.1)*
P_ET_CO_2_ [kPa]	5.5 (.6)	5.6 (.6)	5.6 (.5)	5.5 (.5)	5.5 (.5)	5.5 (.6)
P_ET_O_2_ [kPa]	20.0 (.9)	19.8 (.8)	19.8 (.7)	20.2 (.9)	19.9 (.7)	20.2 (.8)
Intensity rating [%VAS]	–	12 (16)	–	32 (21)	–	71 (20)*
Unpleasantness rating [%VAS]	–	10 (18)	–	25 (25)	–	61 (32)*
Heart rate [min^−1^] (*N* = 15)	68 (11)	67 (10)	69 (9)	67 (12)	68 (11)	69 (11)

*significantly different from unloaded breathing at *p* < .001.

¶significantly different from anticipation unloaded breathing at *p* < .05.

## 3 Results

### 3.1 Behavioural correlation matrix

**Fig. 1:**
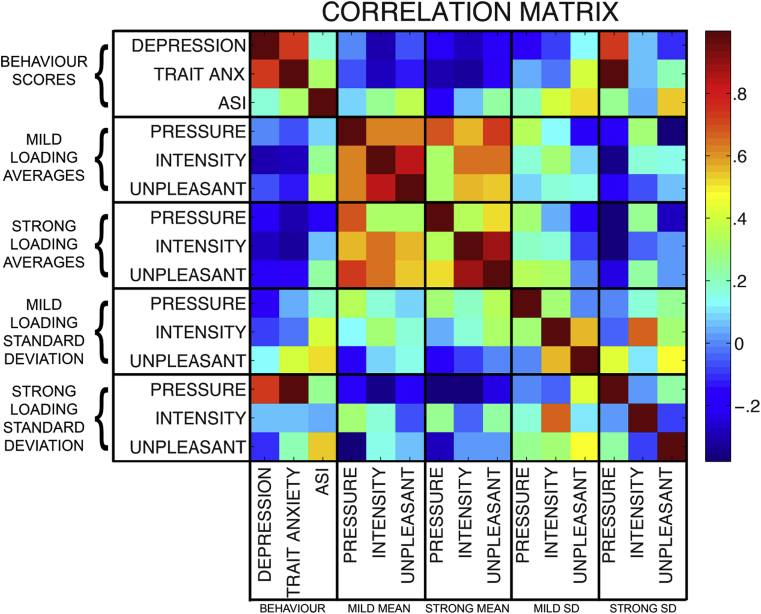
Exploratory, post-hoc full correlation matrix of measured behavioural and physiological variables. Behavioural scores consisted of measures of depression, trait anxiety and anxiety sensitivity index (ASI). Mean and SD measures of mouth pressure, intensity and unpleasantness scores are included for mild and strong resistive loading (breathlessness).

Mean trait anxiety (±SD) was 33.5 (±8.9) points, mean CESD-R 7.3 (±6.3) points and mean ASI 15.4 (±6.7) points. Due to the exploratory nature of this analysis, correlations were not corrected for multiple comparisons (see Supplementary Material for R values and *p* values). Trait anxiety and depression were highly correlated across subjects, but neither correlated with ASI score (Fig. 1). No behavioural scores (depression, trait anxiety or anxiety sensitivity) were found to correlate with mean inspiratory pressure or subjective breathlessness visual analogue scale (VAS) scores (0–100%) of intensity or unpleasantness for either mild or strong breathlessness conditions (Fig. 1). However, when behavioural scores were compared to variability (SD) in physiology and subjective scores, ASI was the only measure that was correlated with *perceptions* (i.e., intensity and unpleasantness) of breathlessness for both mild and strong loading (Fig. 1, Fig. 3). Interestingly, both trait anxiety and depression were strongly correlated with the variation in pressure trace during strong (but not mild) breathlessness, but not subjective scores. The four subjects that were excluded for not forming associations between the cues on the screen and their breathing all had ASI scores within two SDs of the mean (mean excluded: 12.8 ± 8.7 points).

When mean subjective breathlessness scores and physiology were compared, average pressure, subjective intensity and unpleasantness were all strongly correlated during mild breathlessness (Fig. 1). However, during strong breathlessness, intensity and unpleasantness scores became even more strongly correlated while ‘de-coupling’ from measures of inspiratory pressure. Lastly, while variations in intensity and unpleasantness scores were correlated during mild breathlessness, neither was reflective of variation in inspiratory pressure for either level of breathlessness.

### 3.2 Average brain activity during anticipation and breathlessness

**Fig. 2:**
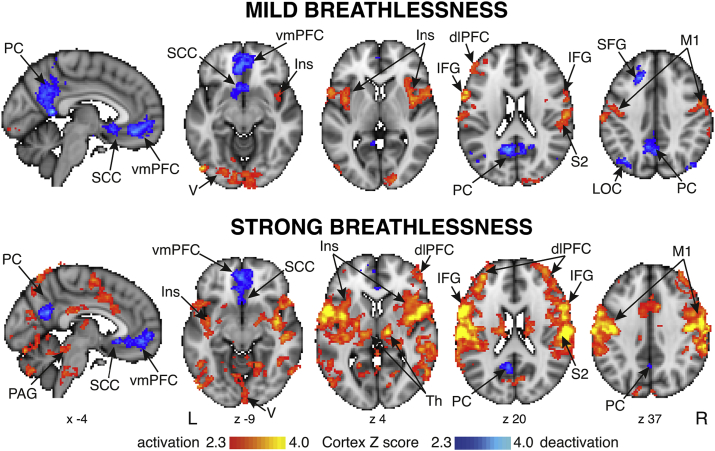
Mean BOLD changes identified during mild and strong breathlessness stimuli. The images consist of a colour-rendered statistical map superimposed on a standard (MNI 2 × 2 × 2 mm) brain. Significant regions are displayed with a threshold *Z* > 2.3, using a cluster probability threshold of *p* < .05 (corrected for multiple comparisons). Abbreviations: vmPFC, ventromedial prefrontal cortex; dlPFC, dorsolateral prefrontal cortex; SCC, subcingulate cortex; Ins, insula; IFG, inferior frontal gyrus; SFG, superior frontal gyrus; M1, primary motor cortex; S2, secondary somatosensory cortex; PC, precuneus; Th, thalamus; LOC, lateral occipital cortex; PAG, periaqueductal gray.

Conditioned associations between visual stimuli and breathlessness stimuli were confirmed prior to scanning in all subjects. Group mean brain activity during anticipation and strong breathlessness has been previously reported (Hayen et al., 2017). No significant mean activity was observed during anticipation of mild breathlessness, and brain activity during mild and strong breathlessness is illustrated in Fig. 2.

### 3.3 Perceptual variation during mild breathlessness

**Fig. 3:**
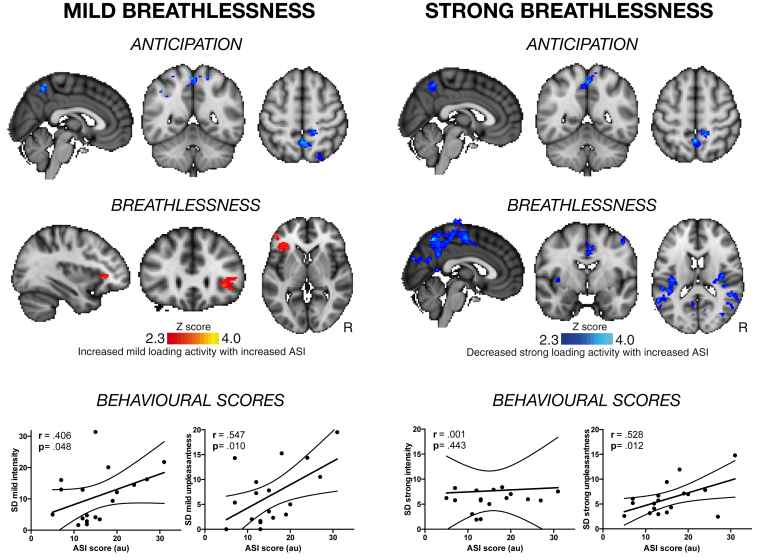
Relationship between perceptual variation, behavioural ASI score and brain activity. Brain activity correlating with ASI score during anticipation (top) and breathlessness (middle) is shown, and significant correlations between behavioural ASI score and perceptual variation (SD) in both intensity and unpleasantness are shown. ASI score negatively correlates with activity in the precuneus cortex during anticipation of both mild and strong breathlessness. Mild breathlessness activity in the anterior insula positively correlates with ASI score, while strong breathlessness activity in the posterior insula, primary motor and sensory cortices, precuneus and posterior cingulate cortex negatively correlate with ASI score. Coloured brain regions represent areas where brain activity correlated with ASI score across subjects. These brain regions were determined using a cluster-forming threshold of *Z* > 2.3, using a cluster probability threshold of *p* < .05 (corrected for multiple comparisons across the whole brain).

During mild breathlessness, the extent of perceptual variation in subjective scores of both breathlessness intensity (*r* = .406, *p* = .048) and unpleasantness (*r* = .547, *p* = .010) were correlated with ASI score. When ASI score was subsequently investigated as a modulator of brain activity during anticipation of mild breathlessness, a negative correlation between ASI and activity in the precuneus cortex was found (Fig. 3). Comparatively, ASI was found to correlate with brain activity in the left anterior insula during perception of mild breathlessness (Fig. 3).

### 3.4 Perceptual variation during strong breathlessness

During strong breathlessness, the extent of perceptual variation in subjective scores of breathlessness unpleasantness was correlated with ASI score (*r* = .528, *p* = .012). Variation in breathlessness intensity no longer correlated with ASI score (*r* = .001, *p* = .443). ASI score was found to negatively correlate with activity in the precuneus cortex during anticipation of strong breathlessness, and in the posterior insula cortex, primary and secondary somatosensory cortices, primary motor cortex, dorsal anterior cingulate cortex, lateral occipital cortex and the precuneus cortex during strong breathlessness (Fig. 3).

## 4 Discussion

In this study we have shown that the greater an individual's anxiety sensitivity index (ASI) score, the greater the variability in breathlessness scores to a set of standardised breathlessness challenges. We then compared anxiety sensitivity across subjects to their functional brain activity during both anticipation and perception of resistive inspiratory loading, in an explorative investigation into the brain-behaviour interface between anxiety sensitivity and the perceptions of breathlessness.

The extent of negative emotions such as anxiety and depression have long been considered potential modulators of perception (Klauenberg et al., 2008, Mallorqui-Bague et al., 2016, Merikle and Joordens, 1997, Phelps et al., 2006, Spinhoven et al., 1997, Tang and Gibson, 2005). However, in healthy populations these scores may not be sensitive enough to identify a potential role in the interoceptive perceptions of breathlessness. In contrast, anxiety sensitivity is a measure of alertness or awareness (not necessarily accuracy) of bodily sensations of anxiety, and worry about the consequences of those sensations (Reiss et al., 1986). Interestingly, in this report we have shown that it is an individual's anxiety sensitivity explains variability in perceived breathlessness more powerfully than generalized anxiety or depression. This attention and vigilance towards bodily sensations might thus render symptoms more ambiguous and susceptible to misinterpretation. Comparatively, trait anxiety and depression instead correlated with mouth pressure variability during strong breathlessness, indicating that participants with high trait anxiety might have modulated their breathing to avert negative sensations, and actively mediated the relationship between symptoms and expected perception.

Numerous previous studies have used a range of breathlessness stimuli to investigate where breathlessness symptoms are processed in the brain (Banzett et al., 1996, Banzett et al., 2000, Banzett et al., 2008, Faull et al., 2016, Hayen et al., 2017, Leupoldt von and Dahme, 2005, Pattinson et al., 2009). What we have learnt is that an extensive network of sensorimotor, affective and stimulus valuation areas are all highly active during breathlessness, as it is such a multi-dimensional experience (De Peuter et al., 2004, Hayen et al., 2013, Herigstad et al., 2011, Schwartzstein et al., 1990). Moving forward, the challenge involves teasing apart where expectations (priors) and neural sensory information meet within this network to allow inference and perception. While studies using conditioned breathlessness cues can help us to understand and isolate the brain networks involved in the generation of priors (Faull and Pattinson, 2017, Faull et al., 2016, Herigstad et al., 2015, Herigstad et al., 2016, Stoeckel et al., 2016), in this report we additionally investigated the perceptual variability around a repeated equivalent stimulus, to probe how behavioural measures of anxiety, depression and anxiety sensitivity may be influencing the distribution of breathlessness scores, and where in the brain this may occur.

**Fig. 4:**
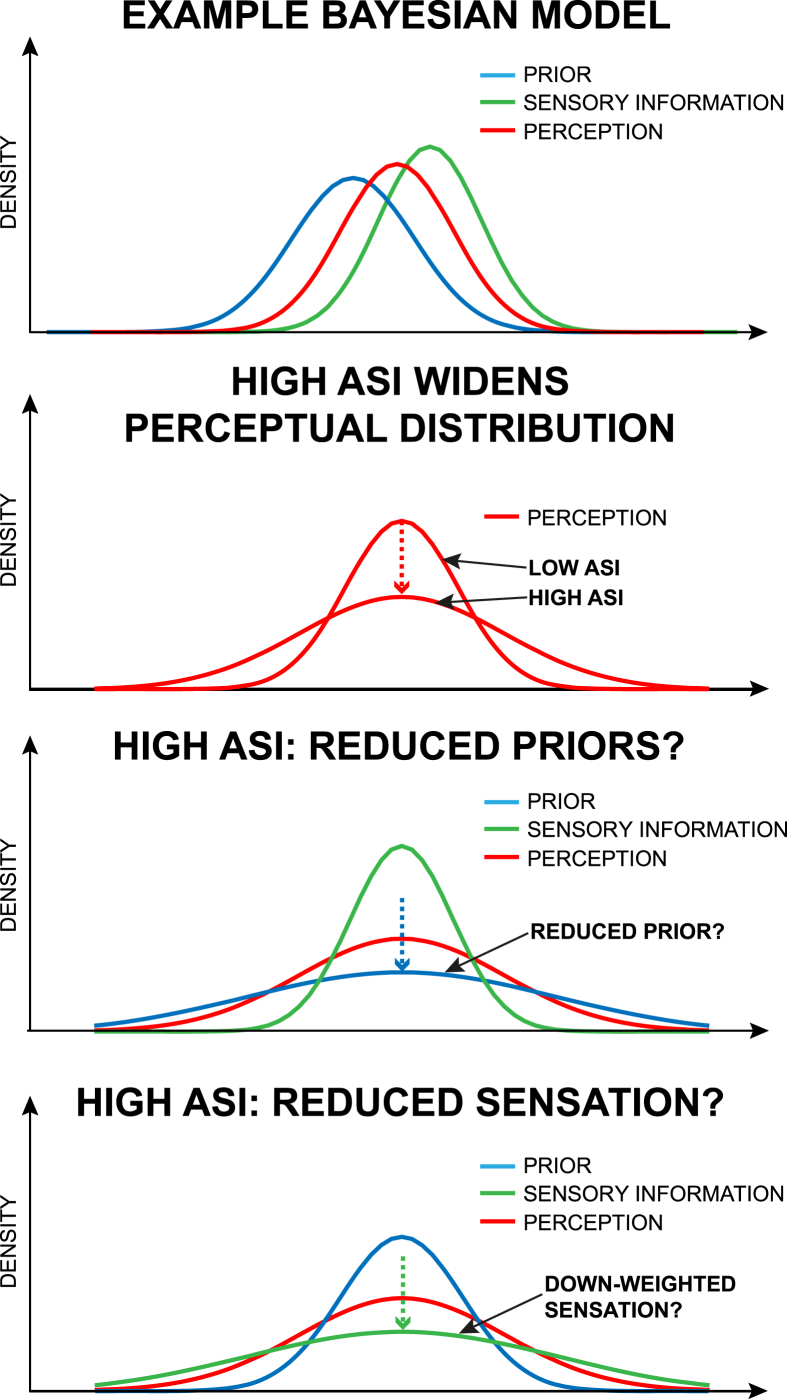
Theoretical possible relationships between ASI and breathlessness perception using a Bayesian framework. Top panel: Example Bayesian parameters. Second panel: Here we show an illustrative representation of the relationship between ASI and perceptual variation, i.e., that high ASI is related to wider perceptual distributions in these subjects. In the bottom two panels, we demonstrate how (within a Bayesian system) this widening of perception may result from either flatter priors (middle panel) or flattening the sensory information from the periphery (bottom panel). This illustration is purely speculative and simply demonstrates that either changing an individual's priors, and/or changing the weight of incoming sensory information may both widen perceptual distributions. Figure adapted from Van den Bergh et al. (2017).

Within the Bayesian framework, the final perception of a symptom such as breathlessness is represented by a set of probable breathlessness perception values (posterior distribution). As this Bayesian system strives for efficiency, it aims to minimize the differences between prior expectations and afferent sensory information (prediction errors) (Friston, 2005). Psychological traits such as anxiety sensitivity could interact with factors such as interoceptive accuracy, or other behavioural properties such as threat detection or catastrophising within an individual. These modulations within the system may either lead to altered expectations, shifted attention or a change in the importance (or gain) assigned to incoming sensory information. All of these possibilities may ultimately lead to changes in this posterior distribution of perceptions (see Fig. 4 for an illustration), and reduced prediction errors (Van den Bergh et al., 2017).

It has been elegantly hypothesized that aspects of this Bayesian framework may be somewhat anatomically distinct within the brain. Specifically, while prior generation may be widespread within a ‘stimulus valuation’ network in the brain (Van den Bergh et al., 2017), interoceptive predictions on the state of the body in the immediate moment from now occur within the deep layers of agranular cortices, such as anterior cingulate cortex and anterior insula (Barrett and Simmons, 2015, Friston, 2005, Shipp et al., 2013, Van den Bergh et al., 2017). These agranular cortices are comprised of many projection neurons that terminate within granular cortices (Barbas, 2015, Barbas and Hilgetag, 2002, Barbas and Rempel-Clower, 1997, Shipp et al., 2013). Granular cortices, such as the primary sensory cortex and posterior insula, consist of well-differentiated layers including granule cells in layer IV that can amplify thalamic sensory inputs (Herkenham, 1980, Larkum, 2012, Staiger et al., 2004), and potential precision cells to tune the gain and alter sensory signals from the periphery (Barrett & Simmons, 2015). Lastly, prediction errors can also be monitored and adjusted by cortico-cortical connections between granular and agranular structures (Barrett & Simmons, 2015), with the addition of input from early threat perception structures such as the midbrain periaqueductal gray (McNally and Cole, 2006, McNally et al., 2011, Roy et al., 2014).

In the current study, participants were conditioned to associate an abstract cue with upcoming mild or strong breathlessness. This learnt association allows the generation of breathlessness expectations, and we were then able to investigate where in the brain the behavioural measure of anxiety sensitivity interacts with brain activity. During anticipation of both mild and strong breathlessness, where prior generation should be at its strongest, anxiety sensitivity inversely correlated with the precuneus cortex, an area of the brain highly implicated in self-reflection and memory retrieval (Mechelli, 2004, Park and Friston, 2013, Vogt and Laureys, 2005). With greater ASI score corresponding to less precuneus activity, it is possible that individuals with high anxiety sensitivity are less reliant on their previous experiences for interoceptive perception.

ASI was also found to correspond to altered brain activity during breathlessness itself. We observed both greater activity in the agranular anterior insula during mild loading, and reduced activity in granular cortices such as the posterior insula and primary sensory cortex (Evrard et al., 2013, Kalia and Mesulam, 1980, Shipp et al., 2013) with greater ASI scores. The anterior insula has been previously implicated in immediate predictions of bodily state within an interoceptive system, and is relatively robust to moment-by-moment prediction errors (Barrett & Simmons, 2015). Additionally, corresponding changes in granular cortices may represent down-modulation of the gain of afferent information. Therefore, it is possible that although those with greater anxiety sensitivity rely less on prior generation to determine their perceptions, the anxiety and attention towards their symptoms may also drives the weight of the Bayesian perception framework away from incoming sensory information and towards transient predictions from agranular cortices, in an attempt to reduce their prediction errors. This disruption from both directions makes this system less robust, and as a result may create the wider posterior perceptual distribution observed in these individuals.

### 4.1 Clinical relevance

The current study has been carried out in healthy volunteers with no history of respiratory disease. Studying healthy populations can aid us in understanding normal variants in physiology, psychology and perception. Still, the challenge remains to apply these concepts to clinical populations. If an individual suffers from chronic breathlessness, they may (over time) alter their priors and thus change their perception. This may result in a shift of the prior further from the neural sensory information (a leftward or rightward shift of the prior illustration in Fig. 4). It remains to be investigated how this change in expectation within the course of chronic disease may be influenced by pre-existing behavioural levels of anxiety and depression, and their potential interactions with anxiety sensitivity. This could help to explain how treatment options such as pulmonary rehabilitation for chronic obstructive pulmonary disease (COPD) may address both the expectations of breathlessness and symptom-related anxiety (Herigstad et al., 2017), and determine in which populations (and under what conditions) such measures would be expected to work most effectively. Using the Bayesian framework to link relevant baseline measures of anxiety, depression, interoceptive attention and sensitivity to neural activation within clinical populations could also help to understand and address maladaptive perceptual differences, e.g., dangerous ‘under-’ and ‘over-’ perception of symptoms in asthma sufferers.

### 4.2 Limitations

This study is a supplementary analysis of previously published work, representing preliminary pilot data in healthy volunteers with small study numbers (*n* = 19) and limited stimulus repetitions (*n* = 4 each for mild and strong breathlessness). Whilst previously published research has demonstrated both improved (Petersen, von Leupoldt, & Van den Bergh, 2015) and worsened (Garfinkel, Manassei et al., 2016) respiratory perceptual accuracy with greater generalised anxiety, the current results showed no effect of trait anxiety on perception. Rather, we have observed a relationship between anxiety sensitivity and perceptual variation. While anxiety sensitivity represents a separate facet of anxiety constrained to bodily sensations (Reiss et al., 1986), numerous other variables may also contribute to differences with previously published results. These factors may include the continuous ratings used in this study compared to categorical ratings used previously (Petersen, Schroijen, Mölders, Zenker, & Van den Bergh, 2014), the relatively low trait anxiety values of the study subjects (mean 34 ± 9 (SD) compared to previous classifications of low (29) and high (55) trait anxiety (Koster, Crombez, Verschuere, Van Damme, & Wiersema, 2006)), and/or the small subject numbers and repeats currently employed.

This study also did not attempt to create a computational Bayesian model to predict breathlessness perception. With the limited measures and post-hoc nature of this study this was not feasible, and meant that we were also unable to estimate the location and shape of the prior in relation to both the sensory observation and resulting perceptual (posterior) distribution. It is possible that anxiety sensitivity, anxiety and/or depression induce a lateral shift of the prior, and our assumed changes in prior shape were inferred from the resulting changes in perceptual variation. It is clear that further work is required to explore the relationship between anxiety sensitivity, prior generation and sensory information, and how this may interact with a broad spectrum of generalized anxiety, to more soundly determine its place within the Bayesian symptom perception framework.

In these preliminary results, Z statistic images were thresholded using clusters determined by *Z* > 2.3 and a (family-wise error (FWE) corrected) cluster significance threshold of *p* < .05. Recent concerns have been raised over cluster-based thresholding when combined with parametric testing within fMRI statistical methods (Eklund, Nichols, & Knutsson, 2016). While FILM-based autocorrelation correction in FSL minimizes inflations in the rate of false positive results, further investigations involving larger sample sizes and resultant increases in statistical power are required for more stringent cluster thresholding, to more robustly interrogate these ideas using fMRI in the future.

## 5 Conclusions and future directions

This short report is a preliminary insight into potential mechanisms of perceptual modulation of breathlessness within the Bayesian framework. Within this framework, the brain integrates prior expectations with afferent sensory information to create breathlessness perception. Behavioural modulators could potentially alter this relationship and influence subsequent perceptual distributions. Here, we have shown that level of anxiety sensitivity is related to variations in breathlessness perception within healthy volunteers, possibly modifying both priors and the gain of afferent sensation to induce greater perceptual variability. Therefore, attention to bodily sensations (ASI) may reduce the robustness of this system in healthy individuals, and increase susceptibility to misinterpretation of breathlessness. Future work on larger cohorts needs to address the relationship between anxiety sensitivity, interoceptive accuracy/confidence and breathlessness perceptions, to investigate how both attention to bodily sensations and interoceptive abilities may interact to adjust the doors of symptom perception.

## Competing interests

KP has acted as a consultant for Nektar Therapeutics. The work for Nektar has no bearing on the contents of this manuscript. KP is named as a co-inventor on a provisional UK patent application titled “Use of cerebral nitric oxide donors in the assessment of the extent of brain dysfunction following injury.
