# Supplementary material for: Breathlessness and the body: Neuroimaging clues for the inferential leap
Source: Cortex. 2017 Oct;95:211–21. doi: 10.1016/j.cortex.2017.07.019 (PMC5637166; doi:10.1016/j.cortex.2017.07.019)
Supplement: Supplementary file 1 [file mmc1.docx]

***Supplementary Material***

**Breathlessness and the body: Neuroimaging clues for the inferential leap**

Olivia K Faull^1,2^, Anja Hayen^1,2^, Kyle T S Pattinson^1,2^

^1^FMRIB Centre and ^2^Nuffield Division of Anesthetics, Nuffield Department of Clinical Neurosciences, University of Oxford, Oxford, UK

**Corresponding author:**

Dr Olivia Faull

Nuffield Department of Clinical Neurosciences

University of Oxford

Oxford, UK

Email: [olivia.faull@ndcn.ox.ac.uk](mailto:olivia.faull@ndcn.ox.ac.uk)

Phone: +44 (0)1865 34544

Fax: +44 (0)1865 23079

*Supplementary Table 1: Correlation coefficient matrix of behavioural and physiological variables presented in manuscript Figure 1. Behavioural scores consisted of measures of depression (CESD-R), trait anxiety (TRAIT) and anxiety sensitivity index (ASI). Mean and standard deviation measures of mouth pressure (PRESS.), breathlessness intensity (INT.) and unpleasantness (UNPL.) scores are included for mild and strong resistive loading (breathlessness).*

|  |  | BEHAVIOUR | | | MILD LOADING MEAN | | | MILD LOADING STD DEV | | | STRONG LOADING MEAN | | | STRONG LOADING STD DEV | | |
| --- | --- | --- | --- | --- | --- | --- | --- | --- | --- | --- | --- | --- | --- | --- | --- | --- |
|  |  | CESD-R | TRAIT | ASI | PRESS. | INT. | UNPL. | PRESS. | INT. | UNPL. | PRESS. | INT. | UNPL. | PRESS. | INT. | UNPL. |
| BEHAV. | CESD-R | 1 | 0.7308 | 0.1723 | 0.0092 | 0.3003 | 0.0681 | 0.2087 | 0.2869 | 0.1649 | 0.1429 | 0.0789 | 0.1319 | 0.7259 | 0.0637 | 0.1117 |
|  | TRAIT | 0.7308 | 1 | 0.3296 | -0.0601 | -0.2762 | -0.1347 | -0.3033 | -0.331 | -0.1528 | 0.0529 | -0.0245 | 0.4061 | 0.9899 | 0.0607 | 0.2207 |
|  | ASI | 0.1723 | 0.3296 | 1 | 0.0831 | 0.2678 | 0.3706 | -0.206 | 0.0659 | 0.2304 | 0.1823 | 0.4056 | 0.5225 | 0.2674 | 0.0361 | 0.5277 |
| MILD MEAN | PRESS. | -0.0092 | -0.0601 | 0.0831 | 1 | 0.6295 | 0.6283 | 0.6958 | 0.5644 | 0.7378 | 0.3349 | 0.1351 | -0.1835 | -0.165 | 0.3074 | -0.3751 |
|  | INT. | -0.3003 | -0.2762 | 0.2678 | 0.6295 | 1 | 0.8458 | 0.3136 | 0.6511 | 0.6463 | 0.1788 | 0.3109 | 0.0897 | -0.3405 | 0.1802 | 0.1447 |
|  | UNPL. | -0.0681 | -0.1347 | 0.3706 | 0.6283 | 0.8458 | 1 | 0.3249 | 0.5623 | 0.5355 | 0.0886 | 0.1661 | 0.1433 | -0.1881 | -0.0593 | 0.0601 |
| MILD STD DEV | PRESS. | -0.2087 | -0.3033 | -0.206 | 0.6958 | 0.3136 | 0.3249 | 1 | 0.3529 | 0.5225 | 0.3072 | 0.0437 | -0.2145 | -0.3561 | 0.2477 | -0.2873 |
|  | INT. | -0.2869 | -0.331 | 0.0659 | 0.5644 | 0.6511 | 0.5623 | 0.3529 | 1 | 0.8787 | 0.185 | 0.1749 | -0.0927 | -0.3774 | -0.0501 | 0.0178 |
|  | UNPL. | -0.1649 | -0.1528 | 0.2304 | 0.7378 | 0.6463 | 0.5355 | 0.5225 | 0.8787 | 1 | 0.3394 | 0.3203 | -0.0078 | -0.2347 | 0.2321 | 0.0144 |
| STRONG MEAN | PRESS. | -0.1429 | 0.0529 | 0.1823 | 0.3349 | 0.1788 | 0.0886 | 0.3072 | 0.185 | 0.3394 | 1 | 0.2912 | -0.0017 | -0.0065 | 0.1745 | 0.2552 |
|  | INT. | -0.0789 | -0.0245 | 0.4056 | 0.1351 | 0.3109 | 0.1661 | 0.0437 | 0.1749 | 0.3203 | 0.2912 | 1 | 0.5526 | -0.0395 | 0.6719 | 0.3012 |
|  | UNPL. | 0.1319 | 0.4061 | 0.5225 | -0.1835 | 0.0897 | 0.1433 | -0.2145 | -0.0927 | -0.0078 | -0.0017 | 0.5526 | 1 | 0.4279 | 0.1115 | 0.467 |
| STRONG STD DEV | PRESS. | 0.7259 | 0.9899 | 0.2674 | -0.165 | -0.3405 | -0.1881 | -0.3561 | -0.3774 | -0.2347 | -0.0065 | -0.0395 | 0.4279 | 1 | 0.0203 | 0.2288 |
|  | INT. | 0.0637 | 0.0607 | 0.0361 | 0.3074 | 0.1802 | -0.0593 | 0.2477 | -0.0501 | 0.2321 | 0.1745 | 0.6719 | 0.1115 | 0.0203 | 1 | -0.0872 |
|  | UNPL. | -0.1117 | 0.2207 | 0.5277 | -0.3751 | 0.1447 | 0.0601 | -0.2873 | 0.0178 | 0.0144 | 0.2552 | 0.3012 | 0.467 | 0.2288 | -0.0872 | 1 |

*Supplementary Table 2: Corresponding matrix of p values for correlation matrix in Supplementary Table 1, presented in manuscript Figure 1. Behavioural scores consisted of measures of depression (CESD-R), trait anxiety (TRAIT) and anxiety sensitivity index (ASI). Mean and standard deviation measures of mouth pressure (PRESS.), breathlessness intensity (INT.) and unpleasantness (UNPL.) scores are included for mild and strong resistive loading (breathlessness).*

|  |  | BEHAVIOUR | | | MILD LOADING MEAN | | | MILD LOADING STD DEV | | | STRONG LOADING MEAN | | | STRONG LOADING STD DEV | | |
| --- | --- | --- | --- | --- | --- | --- | --- | --- | --- | --- | --- | --- | --- | --- | --- | --- |
|  |  | CESD-R | TRAIT | ASI | PRESS. | INT. | UNPL. | PRESS. | INT. | UNPL. | PRESS. | INT. | UNPL. | PRESS. | INT. | UNPL. |
| BEHAV. | CESD-R | 1 | 0.0006 | 0.4942 | 0.971 | 0.226 | 0.7883 | 0.406 | 0.2484 | 0.5131 | 0.5716 | 0.7557 | 0.602 | 0.0006 | 0.8017 | 0.6589 |
|  | TRAIT | 0.0006 | 1 | 0.1817 | 0.8128 | 0.2672 | 0.594 | 0.2211 | 0.1797 | 0.545 | 0.8348 | 0.9232 | 0.0944 | 0 | 0.8108 | 0.3787 |
|  | ASI | 0.4942 | 0.1817 | 1 | 0.7432 | 0.2826 | 0.1301 | 0.4122 | 0.795 | 0.3577 | 0.469 | 0.095 | 0.0261 | 0.2833 | 0.8868 | 0.0244 |
| MILD MEAN | PRESS. | 0.971 | 0.8128 | 0.7432 | 1 | 0.0051 | 0.0052 | 0.0013 | 0.0147 | 0.0005 | 0.1743 | 0.5929 | 0.466 | 0.5129 | 0.2146 | 0.1251 |
|  | INT. | 0.226 | 0.2672 | 0.2826 | 0.0051 | 1 | 0 | 0.2051 | 0.0034 | 0.0038 | 0.4779 | 0.2091 | 0.7233 | 0.1668 | 0.4742 | 0.5668 |
|  | UNPL. | 0.7883 | 0.594 | 0.1301 | 0.0052 | 0 | 1 | 0.1884 | 0.0151 | 0.022 | 0.7266 | 0.5101 | 0.5706 | 0.4548 | 0.8153 | 0.8129 |
| MILD STD DEV | PRESS. | 0.406 | 0.2211 | 0.4122 | 0.0013 | 0.2051 | 0.1884 | 1 | 0.1509 | 0.0261 | 0.215 | 0.8632 | 0.3927 | 0.147 | 0.3216 | 0.2477 |
|  | INT. | 0.2484 | 0.1797 | 0.795 | 0.0147 | 0.0034 | 0.0151 | 0.1509 | 1 | 0 | 0.4624 | 0.4877 | 0.7146 | 0.1226 | 0.8436 | 0.944 |
|  | UNPL. | 0.5131 | 0.545 | 0.3577 | 0.0005 | 0.0038 | 0.022 | 0.0261 | 0 | 1 | 0.1682 | 0.195 | 0.9755 | 0.3484 | 0.354 | 0.9548 |
| STRONG MEAN | PRESS. | 0.5716 | 0.8348 | 0.469 | 0.1743 | 0.4779 | 0.7266 | 0.215 | 0.4624 | 0.1682 | 1 | 0.2411 | 0.9947 | 0.9796 | 0.4885 | 0.3068 |
|  | INT. | 0.7557 | 0.9232 | 0.095 | 0.5929 | 0.2091 | 0.5101 | 0.8632 | 0.4877 | 0.195 | 0.2411 | 1 | 0.0174 | 0.8762 | 0.0023 | 0.2244 |
|  | UNPL. | 0.602 | 0.0944 | 0.0261 | 0.466 | 0.7233 | 0.5706 | 0.3927 | 0.7146 | 0.9755 | 0.9947 | 0.0174 | 1 | 0.0765 | 0.6596 | 0.0507 |
| STRONG STD DEV | PRESS. | 0.0006 | 0 | 0.2833 | 0.5129 | 0.1668 | 0.4548 | 0.147 | 0.1226 | 0.3484 | 0.9796 | 0.8762 | 0.0765 | 1 | 0.9363 | 0.3612 |
|  | INT. | 0.8017 | 0.8108 | 0.8868 | 0.2146 | 0.4742 | 0.8153 | 0.3216 | 0.8436 | 0.354 | 0.4885 | 0.0023 | 0.6596 | 0.9363 | 1 | 0.7308 |
|  | UNPL. | 0.6589 | 0.3787 | 0.0244 | 0.1251 | 0.5668 | 0.8129 | 0.2477 | 0.944 | 0.9548 | 0.3068 | 0.2244 | 0.0507 | 0.3612 | 0.7308 | 1 |
